# A Novel Model of Asymptomatic *Plasmodium* Parasitemia That Recapitulates Elements of the Human Immune Response to Chronic Infection

**DOI:** 10.1371/journal.pone.0162132

**Published:** 2016-09-01

**Authors:** Mary F. Fontana, Alyssa Baccarella, Joshua F. Craft, Michelle J. Boyle, Tara I. McIntyre, Matthew D. Wood, Kurt S. Thorn, Chioma Anidi, Aqieda Bayat, Me Ree Chung, Rebecca Hamburger, Chris Y. Kim, Emily Pearman, Jennifer Pham, Jia J. Tang, Louis Boon, Moses R. Kamya, Grant Dorsey, Margaret E. Feeney, Charles C. Kim

**Affiliations:** 1 Division of Experimental Medicine, Department of Medicine, University of California San Francisco, San Francisco, California, 94143, United States of America; 2 The Burnet Institute, Center for Biomedical Research, Melbourne, Australia; 3 Department of Pathology, Division of Neuropathology, University of California San Francisco, San Francisco, California, 94143, United States of America; 4 Department of Biochemistry and Biophysics, University of California San Francisco, San Francisco, California, 94158, United States of America; 5 EPIRUS Biopharmaceuticals, Utrecht, Netherlands BV; 6 School of Medicine, Makerere University College of Health Sciences, Kampala, Uganda; 7 Division of Infectious Diseases, Department of Medicine, University of California San Francisco, San Francisco, California, 94143, United States of America; 8 Division of Pediatric Infectious Diseases and Global Health, Department of Pediatrics, University of California San Francisco, San Francisco, California, 94143, United States of America; Agency for Science, Technology and Research - Singapore Immunology Network, SINGAPORE

## Abstract

In humans, immunity to *Plasmodium* sp. generally takes the form of protection from symptomatic malaria (i.e., 'clinical immunity') rather than infection ('sterilizing immunity'). In contrast, mice infected with *Plasmodium* develop sterilizing immunity, hindering progress in understanding the mechanistic basis of clinical immunity. Here we present a novel model in which mice persistently infected with *P*. *chabaudi* exhibit limited clinical symptoms despite sustaining patent parasite burdens for many months. Characterization of immune responses in persistently infected mice revealed development of CD4^+^ T cell exhaustion, increased production of IL-10, and expansion of B cells with an atypical surface phenotype. Additionally, persistently infected mice displayed a dramatic increase in circulating nonclassical monocytes, a phenomenon that we also observed in humans with both chronic *Plasmodium* exposure and asymptomatic infection. Following pharmacological clearance of infection, previously persistently infected mice could not control a secondary challenge, indicating that persistent infection disrupts the sterilizing immunity that typically develops in mouse models of acute infection. This study establishes an animal model of asymptomatic, persistent *Plasmodium* infection that recapitulates several central aspects of the immune response in chronically exposed humans. As such, it provides a novel tool for dissection of immune responses that may prevent development of sterilizing immunity and limit pathology during infection.

## Introduction

The *Plasmodium* parasites that cause malaria in humans are noteworthy from an immunological standpoint for their ability to evade sterilizing immunity. In fact, humans in malaria-endemic areas may be infected repeatedly without developing significant ability to prevent blood-stage infection [1]. The natural immunity that does develop in exposed human subjects may be classified into three stages: first, protection from serious disease, which can be fairly rapidly acquired and limits grave clinical symptoms such as severe anemia and cerebral malaria; second, immunity to mild symptomatic disease; and third, partial control of parasite burden, which takes many years and repeated infections to develop [2]. The ability to limit symptomatic malaria disease despite the presence of blood-stage parasites is referred to as clinical immunity.

Clinical immunity to malaria is likely to involve the careful regulation of immune responses to partially control parasitemia while avoiding excessive inflammation, which itself can be pathological and drive clinical symptoms such as fever and anemia [3,4]. Several mechanisms have been proposed to contribute to maintenance of this balance, including increased activity of regulatory T cells [4]; secretion of antibodies against *Plasmodium*-derived toxins [3]; downregulation of Toll-like receptor signaling [5]; production of anti-inflammatory cytokines by activated CD4^+^ T cells [6,7]; and loss of pro-inflammatory, *Plasmodium*-reactive Vδ2^+^ γδ T cells upon repeated exposure to parasites [8]. However, the functional importance of each of these phenomena in limiting pathology in humans has not been defined, and indeed it is difficult to dissect their contributions to clinical immunity in human subjects, where experimental tools are limited.

An additional mechanism by which repeatedly exposed subjects might downregulate pathological immune responses is through exhaustion, an altered differentiation state that develops in T cells chronically exposed to antigen. Described most extensively in CD8^+^ T cells during chronic viral infections, exhaustion involves upregulation of inhibitory surface receptors, including programmed cell death protein 1 (PD-1) and lymphocyte activation gene 3 (LAG-3), followed by progressive loss of T cell effector functions, such as cytokine secretion [9]. In some settings, blockade of exhaustion-associated receptors is sufficient to restore T cell activity and clear chronic infection. Importantly, exhausted T cells are not nonfunctional; there is substantial evidence that they contribute to limiting viral loads during chronic infections that cannot be cleared, while avoiding overwhelming pathology to the host [9,10]. Thus, they have been suggested to be important mediators of a ‘stalemate’ between the host and the pathogen during chronic infection [9].

The *P*. *chabaudi* and *P*. *yoelii* mouse models of blood-stage infection, which recapitulate many aspects of the human immune response during primary uncomplicated *P*. *falciparum* malaria, have greatly informed our knowledge of immune control of *Plasmodium* [11,12]. Evidence for malaria-associated T cell exhaustion has accumulated in recent years [13], beginning with the discovery that *Plasmodium* infection induces upregulation of PD-1 and LAG-3 on CD4^+^ and CD8^+^ T cells in both humans [14–16] and mice [15,17–19]. A role for exhaustion-associated receptors in inhibiting parasite control has been demonstrated in mouse models in which blockade of PD-1 and LAG-3 or deletion of PD-1 accelerated parasite clearance [15,19]. Furthermore, blockade of these receptors was shown to improve secretion of inflammatory cytokines by PBMCs isolated from *P*. *vivax-*infected humans and restimulated *in vitro* [16], providing evidence that T cell exhaustion may limit anti-parasite responses in human subjects.

However, in contrast to both human malaria and to chronic animal models in which T cell exhaustion is associated with failure to clear infection, mice infected with *P*. *chabaudi* and *P*. *yoelii* do achieve sterilizing immunity and are able to efficiently control re-infection with homologous blood-stage parasites [20] despite the presence of exhaustion markers during primary infection [15,17,19]. Thus, it is not clear whether valid parallels can be drawn between the T cell dysfunction observed in mouse models and that occurring in the human host, who can be re-infected multiple times each year [21]. The stark difference between humans and mice in resistance to re-infection has made the mouse model inappropriate for studying mechanisms that enable clinical immunity and limit sterilizing immunity [22]. Without an animal model of asymptomatic *Plasmodium* parasitemia, it has been difficult to interrogate the importance of T cell exhaustion and other immunoregulatory mechanisms in the development of clinical immunity and the disruption of lasting sterilizing immunity.

In this study, we present a novel model of asymptomatic parasitemia in which mice infected with *P*. *chabaudi* sustain patent parasite burdens for many months while remaining apparently healthy. We find evidence for several immunoregulatory mechanisms that may limit pathology and disrupt sterilizing immunity in these mice, including exhaustion of CD4^+^ T cells and production of the regulatory cytokine IL-10. We also show additional parallels between the immune compartments of persistently infected mice and those of chronically exposed human subjects, including increased numbers of B cells expressing the inhibitory marker FCRL5 and a dramatic expansion of nonclassical monocytes, a novel observation that we then corroborate in human cohorts from endemic areas. This work establishes an animal model for further dissection of factors that promote clinical immunity and disrupt sterilizing immunity in chronic settings. Since asymptomatically infected individuals represent an obstacle to the treatment and eradication of many chronic diseases, including malaria [23], a deeper understanding of the underlying immune response is likely to have important implications for human health.

## Results

### Development of a persistent *P*. *chabaudi* infection model

**Fig 1:**
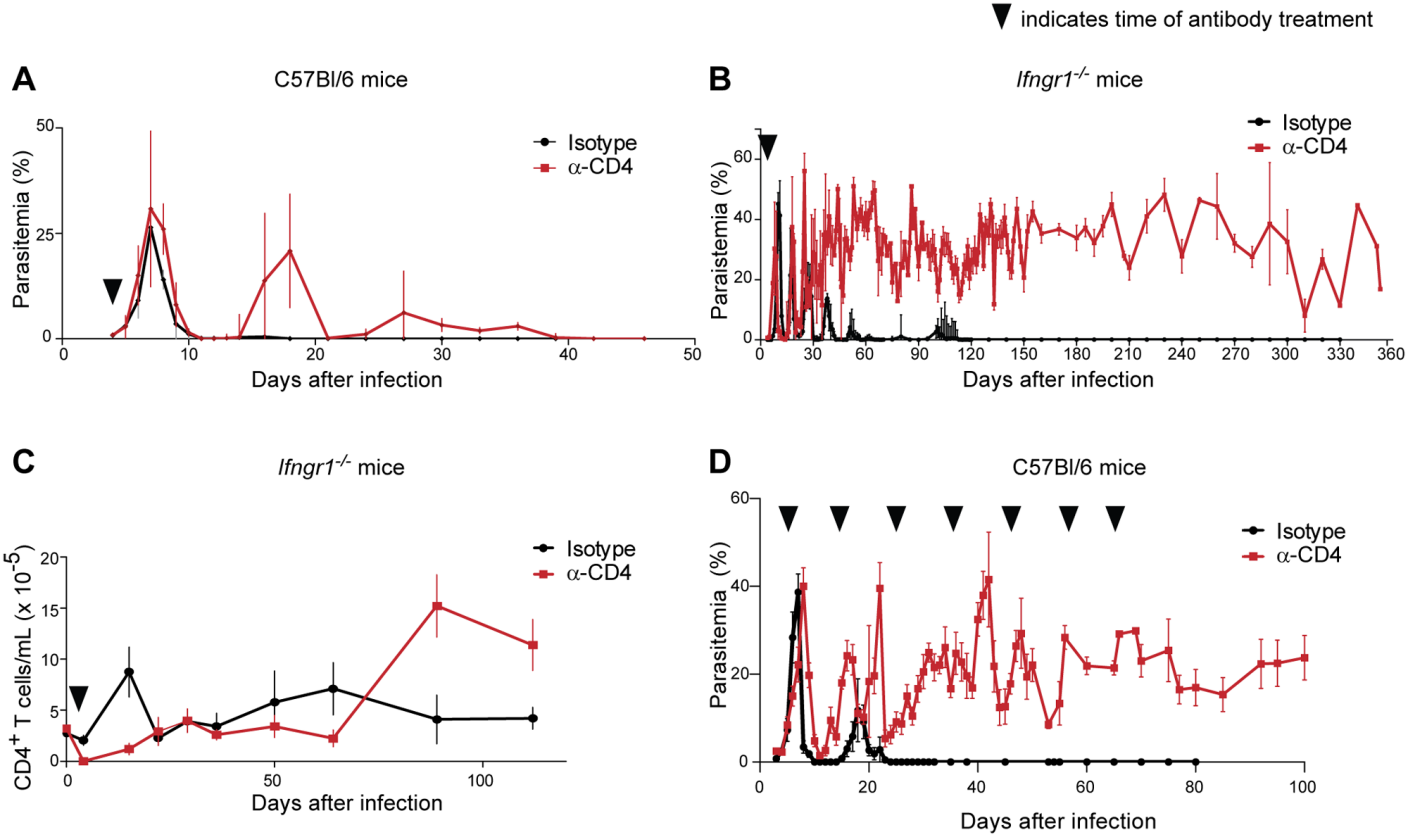
A novel model of persistent, patent *P*. *chabaudi* infection. (A) Wild-type (C57BL/6; B6) mice infected with *P*. *chabaudi* AS were treated 4 d.p.i. with α-CD4 antibody to deplete CD4^+^ T cells, or with an irrelevant isotype control antibody (n = 3 per group). Parasitemia was monitored by thin blood smear. (B) Parasitemia was monitored in *Ifngr1*^*-/-*^ mice infected and treated with α-CD4 (n = 14) or isotype (n = 3) as in (A). (C) *Ifngr1*^*-/-*^ mice were infected and treated as in (B). CD4^+^ T cells were enumerated in blood by flow cytometry (n = 5 control and 15 α-CD4-treated mice). (D) Infected B6 mice (n = 22) were treated through d 64 with α-CD4 or isotype control antibody. Black wedges indicate antibody administration. Means + SEM are shown. In B-D, data shown are pooled from at least three independent biological replicates.

In order to develop an animal model of asymptomatic *Plasmodium* parasitemia, we perturbed components of the immune system in C57BL/6 (B6) mice infected with *P*. *chabaudi* AS, a nonlethal parasite that recapitulates many aspects of human infection with *P*. *falciparum* [12] and elicits a sterilizing immune response in wild-type mice [20,24]. We and others have previously shown that mice lacking a subunit of the interferon gamma (IFN-γ) receptor (*Ifngr1*^*-/-*^) experience higher parasitemias than wild-type mice, but eventually clear infection [25,26]. Similarly, mice transiently depleted of CD4^+^ T cells using an α-CD4 antibody exhibited defective parasite control, but ultimately suppressed the infection to subpatent levels (Fig 1A and [27]). In contrast, transient depletion of CD4^+^ T cells 4 days post-infection (d.p.i.) in *Ifngr1*^*-/-*^ mice resulted in initial dramatic fluctuations in parasite burden, followed by a relatively stable high-level parasitemia (10–50% of RBCs infected) that was sustained for the duration of the observation window (> 300 days; Fig 1B) in the majority of mice (~60%, with ~20% eventually clearing the infection and ~20% requiring euthanasia according to established humane endpoints). We refer to this state as ‘persistent infection’ to distinguish it from previously described models of ‘chronic infection' in B cell-deficient mice, which exhibit long-lasting but very low parasitemia (<1%) [28–30], and from sub-patent parasite levels found in wild-type mice that are in the process of clearing infection [19,31]. The parasitemias observed in persistently infected mice were markedly higher than typical parasite levels found in asymptomatically infected humans, a distinction that has also been noted previously as a general feature of acute mouse models of malaria [22]. Persistently infected mice failed to control their parasitemia despite the complete repopulation of their T cell compartments by 3 weeks post-infection (Fig 1C). Importantly, repeated depletion of CD4^+^ T cells in wild-type mice for approximately the first two months of infection also resulted in stably high parasitemia that continued even after repopulation of the T cell compartment, demonstrating that persistent infection can endure in the presence of a fully intact immune system (Fig 1D and [27,32]). Subsequent experiments to characterize the persistent infection model were performed in both the wild-type and *Ifngr1*^*-/-*^ backgrounds, with great similarity observed between the two genotypes overall.

### Persistently infected mice develop limited clinical symptoms

**Fig 2:**
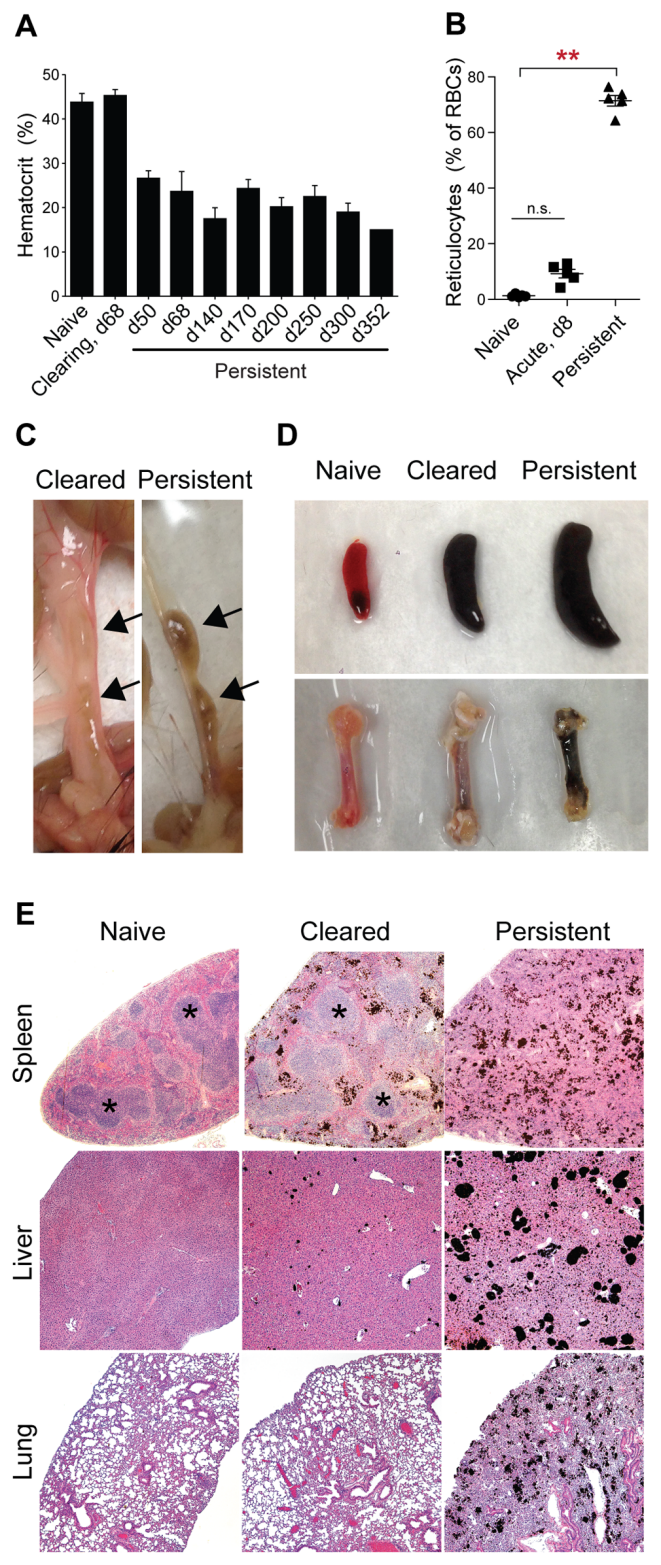
Blood and tissue pathology in persistently infected mice. (A) Hematocrit (mean + SEM) was measured on the indicated days (d) post infection (n = 3–5 mice per group, except n = 1 on day 352). Clearing, *Ifngr1*^*-/-*^ mice infected and treated with control antibody as in Fig 1B. Persistent, *Ifngr1*^*-/-*^ mice infected and treated with α-CD4 antibody as in Fig 1B. (B) Reticulocytes were enumerated as a percentage of total erythrocytes. 8 d.p.i., *Ifngr1*^*-/-*^ mice infected with *P*. *chabaudi* for 8 d. Each dot represents one mouse; means + SEM are shown. **, p < 0.01 by Kruskal-Wallis test with Dunn's post-test. n.s., not significant. (C) Mesenteric lymph nodes (arrows) in cleared and persistently infected mice, 331 d.p.i. (D) Spleens (top) and femurs (bottom) from representative mice, 331 d.p.i. (E) Tissues were excised from the indicated mice 331 d.p.i., processed, and stained with hemotoxylin and eosin. Asterisks mark representative B cell follicles in naive and cleared spleen sections.

To determine whether persistently infected mice exhibited symptoms of acute malaria, we assessed a number of physiological and histological parameters. Persistently infected mice experienced disrupted blood homeostasis, as evidenced by severe anemia (Fig 2A) and reticulocytosis (Fig 2B). They also displayed lymphadenopathy and splenomegaly, disruption of splenic architecture, thickening of the interstitial linings of the lungs, and deposition of dark pigment, likely hemozoin and/or hemosiderin, in the liver, lungs, spleen, bone marrow, and lymph nodes (Fig 2C–2E). A number of these characteristics have been observed in human malaria, most notably anemia, splenomegaly, disruption of splenic architecture, and pigment deposition in the lungs, spleen, and liver [33–36].

**Fig 3:**
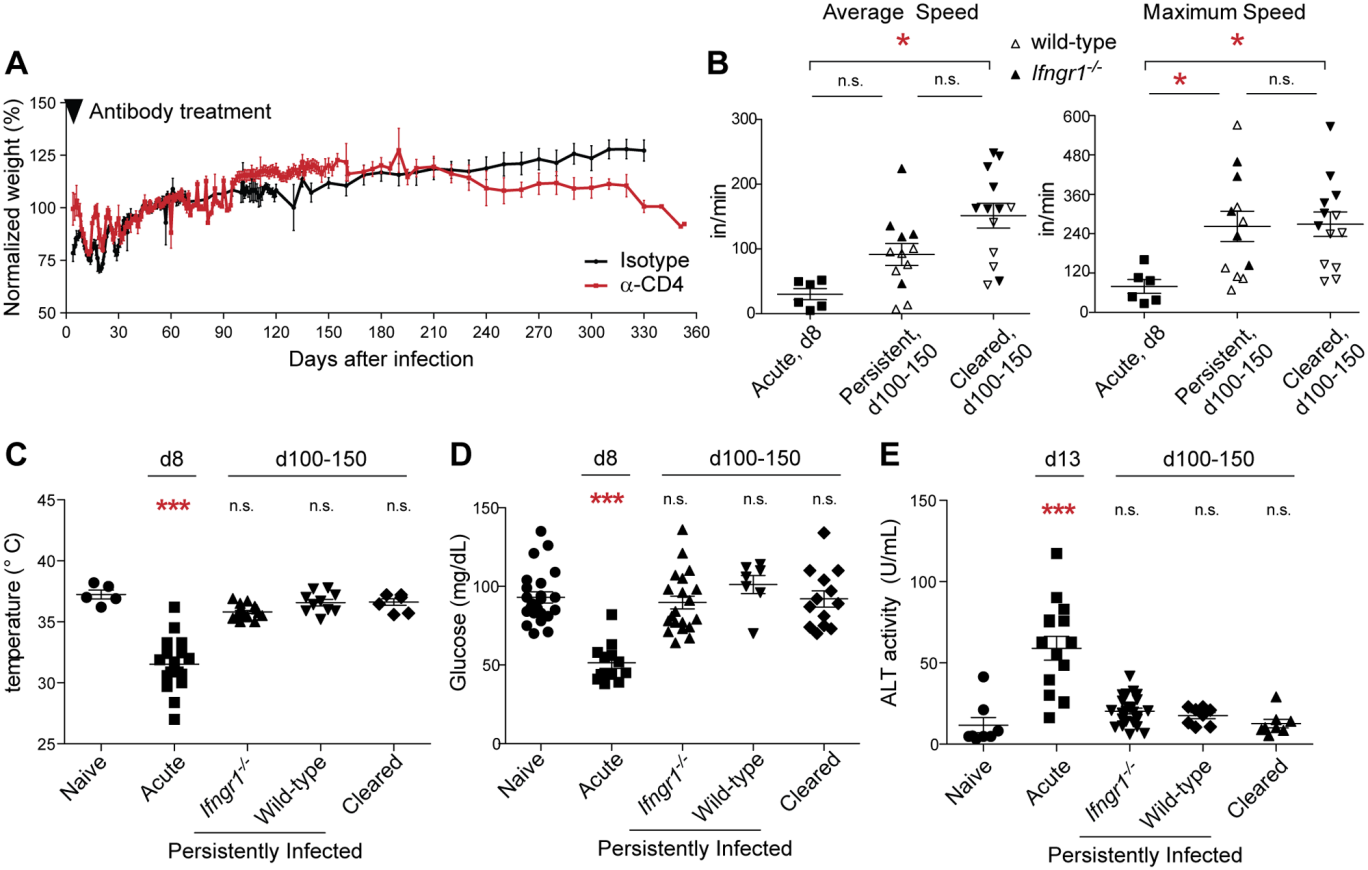
Persistently infected mice display limited clinical symptoms. Mice of the indicated genotypes were infected and treated as in Fig 1B (for *Ifngr1*^*-/-*^) or Fig 1D (for wild-type) to establish persistent infection. (A) The weights of infected *Ifngr1*^*-/-*^ mice are graphed as a percentage of their value 50 d.p.i. (n = 3 isotype-treated, 14 α-CD4-treated). Black wedge indicates time of antibody treatment. (B) Average (left) and maximum (right) mouse activity was measured by open field test. (C) Body temperature, (D) blood glucose, and (E) plasma ALT activity were measured. Acute measurements were taken from *Ifngr1*^*-/-*^ mice 8 d.p.i.; measurements from persistently infected mice were made 100–150 d.p.i. (B, C, D) or 13 d.p.i. (E). In B-E, each point represents an individual mouse; samples were pooled from at least three independently established cohorts. Significance in B-E was determined by Kruskal-Wallis test with Dunn's post-test. In C-E, significance values represent comparison with naive mice. *, p < 0.05. **, p < 0.01. ***, p < 0.001. n.s., not significant.

Despite these gross pathologies, persistently infected mice displayed improved health relative to acutely infected mice. After the acute phase, persistently infected mice recovered weight at a rate similar to age-matched, isotype control-treated mice that cleared infection (referred to as 'cleared'; Fig 3A), and were more active (Fig 3B) and less hypothermic (Fig 3C) than acutely infected mice. Blood glucose levels, which drop sharply during acute infection [37], were normal in persistently infected mice (Fig 3D). In acutely infected mice and humans, elevated plasma levels of the enzyme alanine aminotransferase (ALT) indicate liver damage [34,38]; in contrast, this marker was normal in persistently infected mice (Fig 3E) despite extensive pigment deposition in the liver (Fig 2E). Altogether, a number of clinical symptoms associated with acute malaria—weight loss, low activity, hypothermia, hypoglycemia, and liver damage—were absent or significantly mitigated in persistently infected mice, despite the presence of parasite burdens similar to those observed at the peak of acute infection. The apparent lack of morbidity was not due to the absence of IFN-γ or CD4^+^ T cells in *Ifngr1*^*-/-*^ mice, since clinical symptoms were also absent in persistently infected wild-type mice with fully repopulated T cell compartments (Fig 3B–3E). Thus this infection model allows establishment of long-term patent parasitemia accompanied by limited or no symptomatic disease. To our knowledge, it is the first mouse model of *Plasmodium* infection with these features.

### Persistent infection drives T cell exhaustion

**Fig 4:**
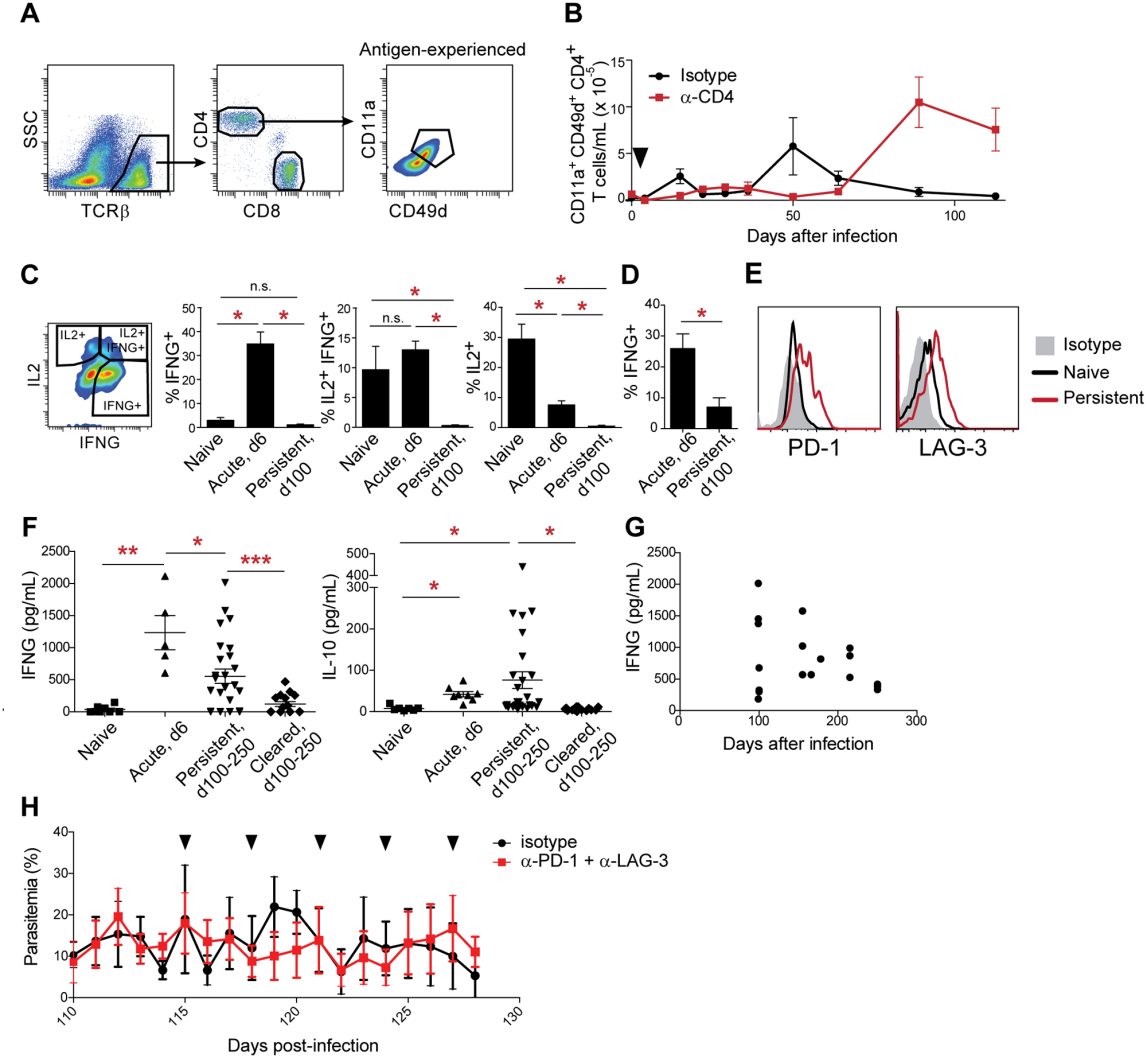
T cell exhaustion in persistently infected mice. (A) Gating strategy to identify antigen-experienced CD4^+^ T cells with the proxy markers CD11a and CD49d. (B) Repopulation of antigen-specific T cells in the blood of infected *Ifngr1*^*-/-*^ mice treated 4 d.p.i. (black wedge) as indicated. Means + SEM are shown (n = 5 control and 15 α-CD4-treated mice, pooled from three independent biological replicates). (C) IL-2 and IFN-γ production was measured by intracellular flow cytometry in blood CD4^+^ T cells from naive, acutely infected (6 d.p.i.) or persistently infected *Ifngr1*^*-/-*^ mice. A representative gating strategy is shown. Means + SEM are shown (n = 5 per group). (D) As in (C), except IFNG^+^ CD4^+^ T cells were measured in wild-type mice. (E) Surface expression of PD-1 and LAG-3 was measured on blood CD4^+^ T cells from *Ifngr1*^*-/-*^ mice by flow cytometry. (F) Levels of IFN-γ and IL-10 were measured in plasma. Acute samples were taken 6 d.p.i. Each point represents one mouse; data are pooled from at least three independent experiments and include samples from both wild-type and *Ifngr1*^*-/-*^ mice, collected from 100–250 d.p.i.. Statistical significance in (D and F) was determined by Kruskal-Wallis test with Dunn's post-test. (G) Plasma IFN-γ levels in persistently infected mice, graphed by time post-infection. (H) Persistently infected wild-type mice (n = 4 per group) were treated every 3 d with 300 μg each α-PD-1 and α-LAG-3 or with isotype control antibodies. Black wedges indicate treatment days. *, p < 0.05. **, p < 0.01. ***, p < 0.001. n.s., not significant.

The sustained parasite burdens and mild clinical symptoms observed in persistently infected mice suggest a state of dampened immune activation in response to parasite antigens. Lacking tools to label *Plasmodium*-specific T cells, we identified antigen-experienced T cells in persistently infected mice by their upregulation of the activation-associated integrins CD11a and CD49d, an approach that has been validated in acute malaria [15,39] (Fig 4A). We found that antigen-experienced CD4^+^ T cells expanded and were retained at high numbers in persistently infected mice, whereas control mice experienced expansion of these cells followed by contraction as the infection was cleared (Fig 4B). Antigen-experienced cells from acutely infected (6 d.p.i.) mice produced robust amounts of the pro-inflammatory cytokine IFN-γ, as well as the T cell growth factor IL-2, upon restimulation with PMA and ionomycin *ex vivo*, whereas antigen-experienced T cells from mice persistently infected for > 100 d produced very little cytokine, regardless of whether the mice were *Ifngr1*^*-/-*^ (Fig 4C) or wild-type (Fig 4D). They also expressed increased levels of the inhibitory receptors PD-1 and LAG-3 (Fig 4E), indicative of T cell exhaustion. Kinetic analysis showed gradual upregulation of PD-1 and LAG-3, and a concurrent decrease in cytokine production, occurring between approximately 50 and 100 d.p.i. with the timing varying between individual mice (data not shown). Plasma IFN-γ was decreased in persistently infected mice relative to acutely infected mice, whereas the anti-inflammatory cytokine IL-10 was elevated, indicating a regulatory, rather than inflammatory, cytokine milieu (Fig 4F). Plasma IFN-γ levels were inversely correlated with time post-infection in persistently infected mice, demonstrating a progressive decrease in production of this cytokine over time (Fig 4G).

A previous report demonstrated that clearance of acute infections with *P*. *yoelii and P*. *chabaudi* could be enhanced by treatment with antibodies to LAG-3 and PD-L1, a ligand for PD-1 expressed on myeloid cells [15]. However, blockade of PD-1 and LAG-3 did not enhance restriction of parasitemia in persistently infected wild-type or *Ifngr1*^*-/-*^ mice (Fig 4H and data not shown). This result suggests the possibility that in addition to T cell exhaustion, other factors that prevent clearance may remain to be discovered in this model.

### Persistent infection drives expansion of FCRL5-expressing B cells

**Fig 5:**
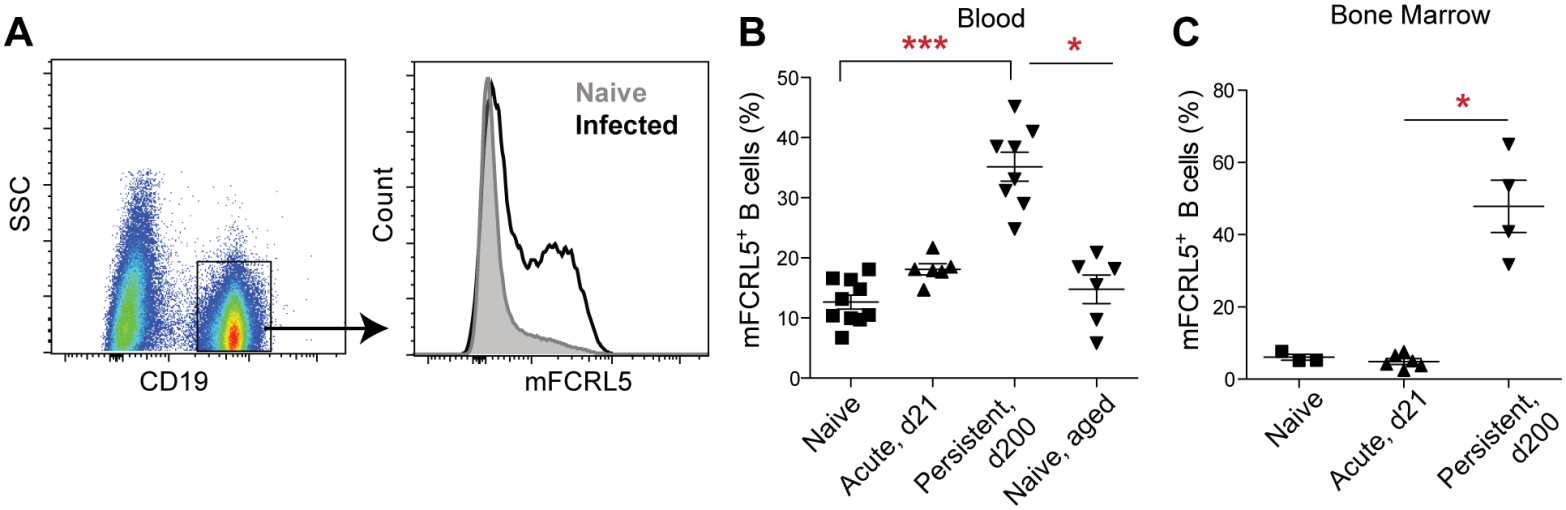
Increased FCRL5^+^ B cell populations in infected mice. (A) Gating strategy and histogram plot showing FCRL5 expression on CD19^+^ B cells from representative naive and persistently infected mice. (B, C) The frequency of FCRL5^+^ B cells was quantified as a percentage of total B cells in the blood (B) or bone marrow (C) of mice that were uninfected (naive), infected for 21 days (acute), or persistently infected for 200 days (persistent). Eight-month-old naive mice (naive, aged) were also assessed as age-matched controls for persistently infected mice. Each symbol represents an individual mouse; results are representative of four (B) or two (C) independent experiments. *, p < 0.05; ***, p < 0.001 by Kruskal-Wallis test with Dunn's post-test.

In order to test whether persistent infection elicits additional immune responses that resemble those occurring in naturally exposed humans, we examined other leukocyte populations. Recently, several studies have highlighted a subset of atypical memory B cells (atMBCs) that expands in humans with chronic *Plasmodium* exposure [40–42]. Unlike classical memory B cells, atMBCs lack expression of CD21 and CD27, but do express the inhibitory receptors FCRL3 and FCRL5 [42]. In the mouse, naïve and memory B cell markers are not well defined, and CD27 is not an appropriate marker for memory cells, as it is in humans [43]. However, mice do possess a gene, *Fcrl5*, that bears homology to both FCRL3 and FCRL5 in humans, and has been reported to have similar expression patterns to human FCRL3 [44]. Therefore, we assessed mouse FCRL5 (mFCRL5) expression on B cells in naive and infected mice (Fig 5A). Because B cells resembling atMBCs also accumulate in aged mice and humans [45], we additionally examined naive mice that were age-matched to persistently infected mice, which were ~ 3 months older than the acutely infected controls in this experiment. Interestingly, we found that both acute and persistent infection with *P*. *chabaudi* resulted in expansion of mFCRL5^+^ B cells in the blood, but that frequencies of mFCRL5^+^ cells were significantly higher in persistently infected mice than in acute mice (Fig 5B). Furthermore, persistent, but not acute, infection caused an increase in mFCRL5 expression on B cells in the bone marrow (Fig 5C), where long-lived plasma cells reside [46]. We saw no increase in FCRL5^+^ B cells in the blood of aged naive mice, indicating that expansion of this population is due to infection rather than age (Fig 5B). These results reveal an additional similarity between the immune profiles of persistently infected mice and those of chronically exposed humans.

### Striking expansion of nonclassical monocytes in mice with persistent *P*. *chabaudi* infection

**Fig 6:**
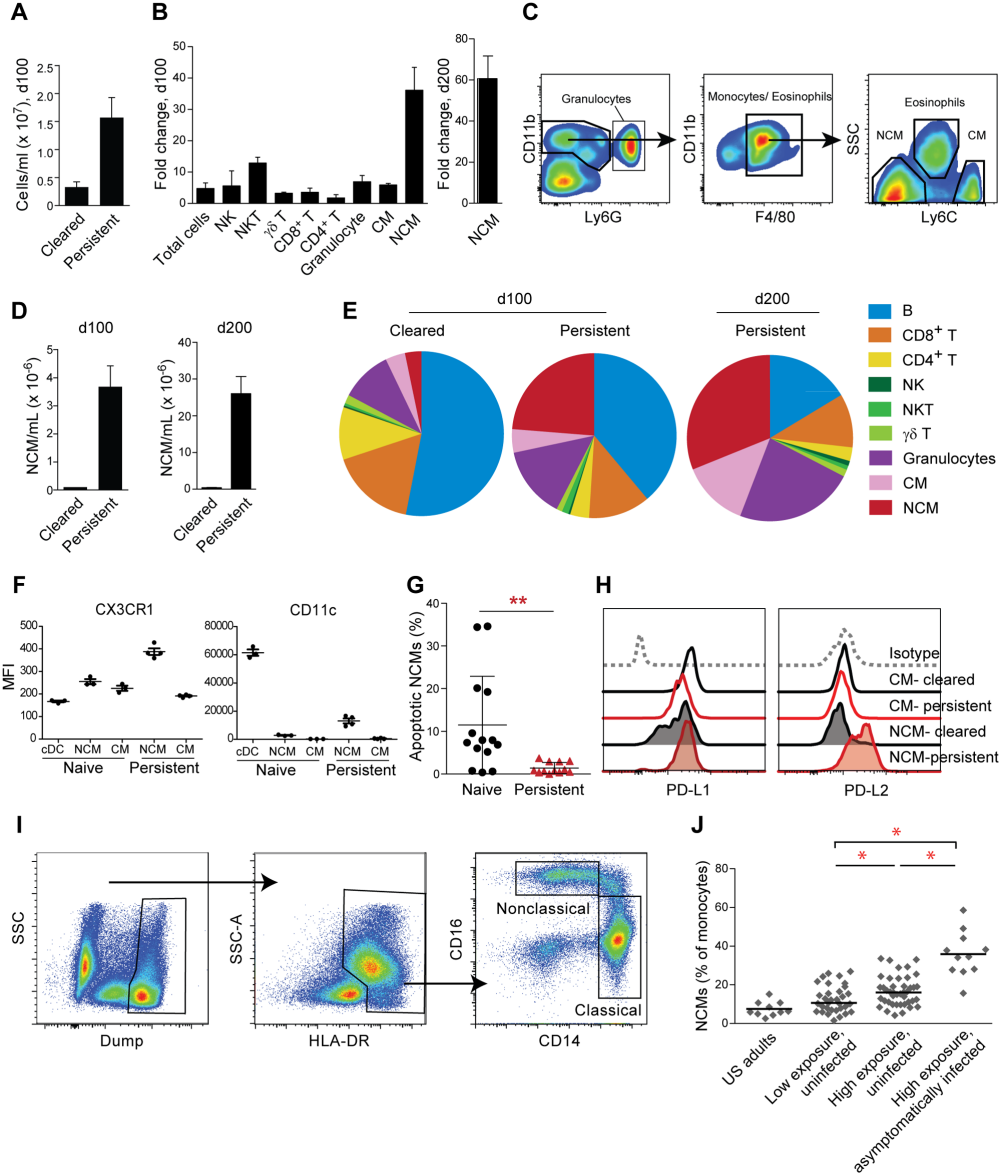
Expansion of nonclassical monocytes in persistent infection and in chronically exposed humans. (A) Leukocytes were quantified in the blood of cleared and persistently infected mice 100 d.p.i. Means + SEM are shown (n = 4 per group). (B) The abundance of each blood leukocyte subset is expressed as the fold increase in persistently infected mice over cleared mice. The sampling timepoint is indicated on the *y*-axis. Means + SEM are shown (n = 4 per group). (C) Gating strategy for classical (Ly6C^hi^) and nonclassical (Ly6C^lo^) monocytes. (D) Blood concentration (mean + SEM) of NCMs in cleared (n = 3) and persistently infected (n = 7) mice. Note different scales on left and right graphs. (E) Frequencies of blood leukocyte subsets. Average values from 3 cleared and 7 persistently infected mice are shown. NK, natural killer cell. NKT, natural killer T cell. CM, classical monocyte. NCM, nonclassical monocyte. (F) Expression of CX3CR1 (left) and CD11c (right) was assessed by flow cytometry on the indicated populations. Each symbol represents one mouse; bars show mean +/- SD. (G) Apoptotic NCMs were quantified in naive and persistently infected mice through flow cytometry. Each point represents one mouse. Means + SEM of four pooled biological replicates are shown. **, p < 0.01 by Mann-Whitney test. (H) Expression of PD-L1 and PD-L2 was assessed on the indicated monocyte populations. Plots from one representative mouse of at least five replicates are shown. (I) Gating strategy for human NCMs. (J) NCM frequencies were assessed in PBMCs by flow cytometry. Low-exposure subjects were from Walakuba, Uganda (EIR = 3.8); high-exposure subjects were from Nagongera (EIR = 125). Each point represents an individual subject. *, p < 0.05 by Wilcoxon. Statistical comparisons to U.S. adults were all significant, but are omitted for clarity.

We continued to examine additional immune populations by quantifying the absolute sizes of individual leukocyte populations in the blood. Persistent infection caused a significant increase in the total number of blood leukocytes (Fig 6A), with numbers of most individual leukocyte subsets examined increasing five- to ten-fold relative to controls in mice persistently infected for 100 days (Fig 6B). However, the most dramatic increase was in the number of nonclassical monocytes (NCMs, defined as CD11b^+^ F4/80^+^ Ly6C^lo^ SSC^lo^ Ly6G^-^; Fig 3C and [47]), which increased by thirty-fold after 100 days of infection and sixty-fold by day 200 (Fig 3B and 3D). In comparison, Ly6C^hi^ classical monocytes (CMs) expanded approximately six-fold by day 100 (Fig 6B). The frequency of NCMs also increased, from < 5% of blood leukocytes in naive or cleared mice to > 25% in persistently infected mice; strikingly, by day 200, NCMs were the predominant leukocyte subset in the circulation (Fig 6E). NCMs expanded to a similar extent in both wild-type and *Ifngr1*^*-/-*^ mice with persistent infections, indicating that this phenomenon is not due to the absence of IFNG (data not shown). NCMs in infected mice robustly expressed the nonclassical subset marker CX3CR1, confirming the identity of this monocyte population; in addition, they displayed modest expression of CD11c (Fig 6F). The increase in NCM numbers appeared to be driven by reduced turnover of this population, since we observed a significant decrease in the frequency of apoptotic NCMs in persistently infected mice relative to naïve mice, as measured by annexin V and propidium iodide labeling (Fig 6G). Interestingly, NCMs from persistently infected mice expressed high levels of the immunosuppressive markers PD-L1 and PD-L2, which can promote T cell exhaustion through ligation of PD-1. In contrast, CMs from persistently infected mice expressed PD-L1, but not PD-L2 (Fig 6H).

### Expansion of NCMs in humans with asymptomatic *Plasmodium* parasitemia

To our knowledge, preferential expansion of NCMs has not been reported in the context of human *Plasmodium* infection, although it has been observed in HIV and other chronic infections [48–50]. To test whether such an expansion occurs, we examined the frequency of CD14^lo^ CD16^+^ monocytes (Fig 6I), corresponding to the murine Ly6C^lo^ NCM subset [47,51], in Ugandan children living in areas of low and high malaria transmission intensity [21]. Uninfected children from a region of high malaria transmission (Nagongera subcounty; annual EIR = 125 infectious bites per year [52]) had significantly higher NCM frequencies than an age-matched cohort of uninfected children from a low-transmission area (Walakuba subcounty; annual EIR = 3.8), suggesting that chronic exposure may drive NCM expansion in humans as well as mice. NCM frequencies were further increased in highly exposed children with asymptomatic parasitemia at the time of sampling, providing additional correlative evidence for a link between expansion of this monocyte subset and the presence of an asymptomatic *Plasmodium* infection (Fig 6J). These results underline yet another immunological parallel between the persistent infection model and human infection, and validate the model as a mechanism for generating insights into clinical immunity that can be subsequently examined in human infection settings.

### Persistent infection abrogates subsequent protective immunity

**Fig 7:**
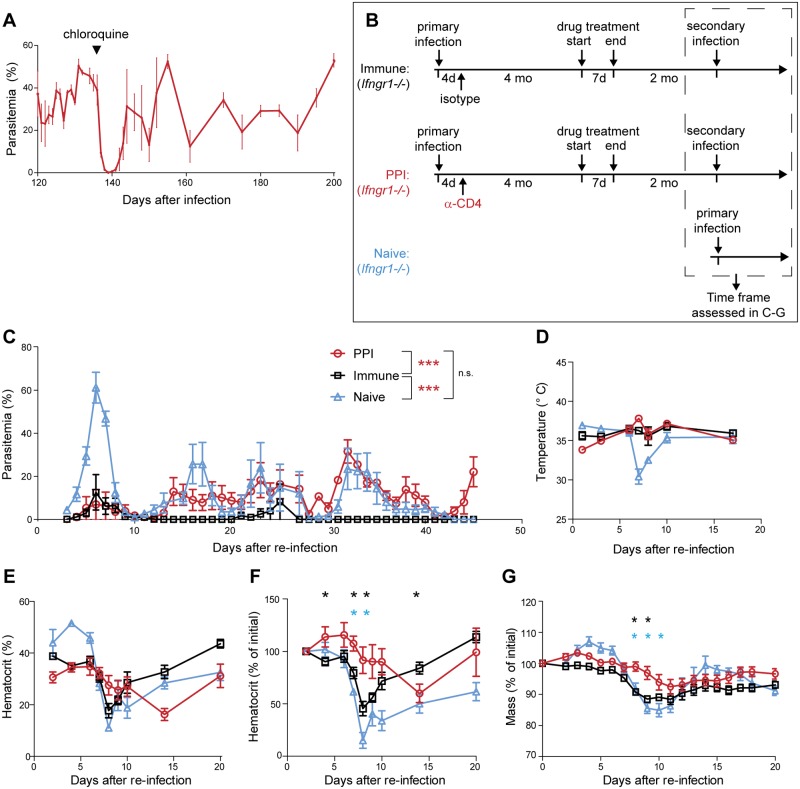
Previously persistently infected mice are not immune to secondary challenge. (A) Persistently infected *Ifngr1*^*-/-*^ mice were treated once with a subcurative dose of chloroquine (black wedge). Parasitemia was monitored during and after treatment. (B-G) *Ifngr1*^*-/-*^ mice were infected and treated with α-CD4 antibody 4 d.p.i. to establish persistent infection, or with an isotype control antibody. After 4 months, all mice were treated with pyrimethamine to clear infection and rested for two months. Isotype-treated mice (immune) and previously persistently infected mice (PPI) were then re-infected with *P*. *chabaudi*. Additional naive *Ifngr1*^*-/-*^ mice were also infected at this time to serve as non-immune controls. (B) Experimental layout. (C) Parasitemia, (D) body temperature, (E, F) hematocrit and (G) weight were monitored at the indicated timepoints. In (F, G), measurements are expressed as a percentage of their value on the day of re-infection. Data shown are means + SEM taken from one of two independent experiments (n = at least 5 for each group in each experiment). *, p < 0.05; ***, p < 0.001 by Mann-Whitney test. n.s., not significant. In F and G, black asterisks denote significant comparisons between immune and PPI groups; blue asterisks denote significant comparisons between PPI and naive groups.

In a previously published model of chronic *P*. *chabaudi* infection in the B cell-deficient mouse, CD4^+^ T cells and other immune mediators resolve acute infection and control chronic parasitemia to < 1% indefinitely [28–30]. In contrast, persistently infected mice in this study’s model exhibit parasitemias of 10–50% for many months, suggesting that immune-mediated restriction of parasite burden is significantly impaired. To better characterize the extent to which immune control is compromised, we first assessed whether persistent parasitemia levels represent a state of homeostasis between parasite replication and immune restriction. Persistently infected mice were injected with a single sub-curative dose of chloroquine, which transiently suppressed parasitemia to nearly undetectable levels. Despite the near disappearance of parasites from the circulation, parasites rebounded to pre-treatment levels within days (Fig 7A), suggestive of a general lack of immunological restriction of parasite replication.

We turned finally to the question of sterilizing immunity following persistent infection, considering the possibility that immune control might continue to be compromised upon clearance and secondary challenge. As noted above, humans can be infected repeatedly with *Plasmodium* sp. without developing sterilizing immunity [1,21], whereas mice acutely infected with *P*. *chabaudi* or other rodent-adapted strains are immune to homologous blood-stage re-infection for many months [20,24]. To examine development of protective immunity, we treated matched cohorts of persistently infected or cleared *Ifngr1*^*-/-*^ mice with an antimalarial drug approximately 4 months post-infection in order to completely eliminate parasites. Two months after drug treatment, mice were re-infected with *P*. *chabaudi*; concurrently, an additional group of naive *Ifngr1*^*-/-*^ mice was infected to serve as controls that completely lacked pre-existing immunity (Fig 7B).

Consistent with previous reports [53,54], naive *Ifngr1*^*-/-*^ mice developed high parasitemia that recrudesced multiple times but was controlled to sub-patent levels by 40–50 days post-infection (Fig 7C). In contrast, mice that had been previously infected and cleared naturally were able to efficiently control parasite growth, exhibiting significantly lower peak parasitemias and more rapid clearance relative to naive mice; henceforth we refer to these as immune mice. We note that they did develop more patent parasitemia than has been previously reported in immunized wild-type mice [24], perhaps indicating a partial requirement for IFN-γ in development of anti-parasite immunity. Strikingly, although previously persistently infected (PPI) mice initially controlled re-infection as well as immune mice, beginning approximately two weeks after re-infection they exhibited sustained levels of parasitemia more similar to those seen in naive mice after an initial infection. Furthermore, whereas naive mice ultimately cleared infection to sub-patent levels, PPI mice re-established persistent infection, failing to clear patent parasitemia for the duration of the observation window (Fig 7C). Thus, a previous persistent infection disrupts development of sterilizing immunity to secondary challenge following pharmacological clearance.

### Previously persistently infected mice are protected from clinical symptoms upon re-infection

We were interested in whether clinical symptoms would be diminished in PPI mice after secondary challenge, as they are during primary infection (Fig 3A–3E). In contrast to naive mice, both immune and PPI mice were protected from hypothermia during the peak of infection (Fig 7D). PPI mice had lower initial hematocrits relative to naive and immune mice, making it difficult to directly compare infection-induced anemia between treatment groups (Fig 7E). However, when hematocrits were normalized to their starting (day-of-re-infection) values, it became apparent that PPI mice experienced a significantly smaller decrease in hematocrit relative to both naive and immune mice (Fig 7F). Additionally, PPI mice lost less weight during the peak of infection than both the naive and immune groups (Fig 7G). Taken together, these data suggest that despite their inability to control parasitemia during a secondary challenge, PPI mice are better able to mitigate infection-associated pathology than naive mice facing acute infection, and fare even better in some parameters than mice with sterilizing immunity.

## Discussion

To our knowledge, the model presented here is the first in which mice bearing the full array of immune subsets fail to develop sterilizing immunity to re-infection with *Plasmodium*. This, combined with numerous similarities between immune responses in persistently infected mice and chronically exposed and/or asymptomatically infected humans, is likely to make this system a powerful tool to further our understanding of the immunological underpinnings of asymptomatic infection. The model recapitulates several important aspects of naturally acquired clinical immunity in humans, including patent parasitemia, prolonged antigen exposure, and failure of sterilizing immunity coupled with limited or absent clinical symptoms.

Mice in this model are continuously exposed to antigen, a situation that may mimic conditions in highly endemic regions of Africa where individuals can receive over three infectious mosquito bites per day [55]. Importantly, several known elements of the human immune response to chronic *Plasmodium* infection are replicated in persistently infected mice, including appearance of exhausted CD4^+^ T cells [13], increased production of IL-10 [6,7,56], and expansion of mFCRL5-expressing B cells ([40–42]). Thus, although the persistent infection model resembles commonly employed acute mouse models with regard to parasite burden, which is significantly higher than in a typical human host, it represents a significant advance over these other systems in modeling the immunological parameters of repeated human infection. In particular, the observed disruption of sterilizing immunity following persistent infection leads us to speculate that this model has great potential for investigating mechanisms that may inhibit sterilizing immunity in chronically exposed humans. As a proof of this concept, we confirmed that one novel finding from our mouse model—the robust expansion of NCMs in asymptomatically infected mice—also occurs in humans with chronic *Plasmodium* exposure and is even more pronounced in humans with concurrent asymptomatic infection. Additionally, since FCRL3 and FCRL5 expression have been associated with B cell dysfunction in humans [41,42], our B cell data are consistent with the notion that persistent infection may perturb normal B cell development and/or function, especially in the critical bone marrow niche.

We found clear evidence of CD4^+^ T cell exhaustion in our model, paralleling results from both acute mouse models and human infection. However, it is notable that in contrast to acute infection [15], blockade of PD-1 and LAG-3 did not promote parasite clearance in this model. This result suggests that additional, redundant suppressive mechanisms may inhibit control of parasitemia in persistently infected mice. Further research will focus on identification of these host and/or parasite factors that disrupt sterilizing immunity; characterization in the mouse model must then be followed by corroborative studies in humans. Additionally, it will be of interest to screen for pathways that promote clinical immunity, which may be considered a form of host tolerance--defined here as limitation of pathology [57,58].

Tolerance of *Plasmodium* infection is generally considered beneficial at the individual level, as it protects the infected host from morbidity and mortality. However, recent studies in humans suggest that asymptomatic parasitemia is not in fact benign [59]. Asymptomatically infected children have been found to possess cognitive defects, lower hemoglobin levels, and higher indicators of inflammation than uninfected children [60,61]. Similarly, persistently infected mice in our model were severely anemic and had lower average activity than cleared mice. Thus, even in the absence of overt illness, sustained *Plasmodium* parasitemia incurs subtle but measureable fitness costs on both mice and humans, making asymptomatic parasitemia a concern for public health. It is also problematic from an eradication perspective: since asymptomatically infected people rarely seek treatment, they constitute a reservoir of parasites from which non-immune individuals, especially children, can continually be infected [23]. Therefore, clinical immunity may represent a significant obstacle to disease control, one with which the field of epidemiology is beginning to grapple [62].

Also importantly for public health, induction of immune exhaustion or tolerance to *Plasmodium* antigens may affect the efficacy of candidate vaccines, which can exhibit strong immunogenicity in naive American adults [63] but subsequently show only weak immunogenicity in adults in endemic areas [64]. Although tolerance has not been demonstrated to cause such vaccine failures, the implications of such a link are significant: first, many potentially effective vaccines might have been inappropriately eliminated from candidacy; and second, testing vaccines on semi-immune adults may be a poor indicator of how a vaccine candidate will perform in the relevant target population—i.e., children who have experienced less exposure to parasites.

Finally, we consider the potential of our new mouse model to address more general but equally important questions about immune regulation in chronic infection. A unique strength of this model is that infection persists stably and indefinitely, but can be cured easily and rapidly with drugs. Therefore it offers an ideal setting in which to study recovery of immune responses, such as reprogramming of dysfunctional T cells, following cure of chronic infections. This area of research has become highly relevant to human health due to recent advances in curative therapies for chronic diseases previously considered incurable [65,66]. Thus, in addition to its insights for anti-malaria immunity, this study establishes a versatile tool for studying diverse aspects of immune responses and recovery during and after chronic infection.

## Materials and Methods

### Ethics statement for human subject research

The portion of this study employing human samples received ethical approval from the Makerere University School of Medicine Research and Ethics Committee, the London School of Hygiene and Tropical Medicine Ethics Committee, the Uganda National Council for Science and Technology, and the University of California, San Francisco Committee on Human Research. A parent or guardian of all participants provided written informed consent.

### Mice

C57BL/6 (B6) mice were from the National Cancer Institute (NCI). *Ifngr1*^*-/-*^ mice on the B6 background were obtained from Jackson Laboratories. Mice were kept on a 12 hour light-dark cycle under specific pathogen free conditions. All mouse work was conducted with the approval of the UCSF Institutional Animal Care and Use Committee (Protocol AN107004) in accordance with the guidelines of the NIH Office of Laboratory Animal Welfare. Infected mice were monitored daily and were euthanized if they failed to display a righting response, according to approved institutional humane endpoint criteria. Approximately 20% of mice treated to establish persistent infection reached humane endpoint criteria and were euthanized. Euthanasia was performed by carbon dioxide inhalation followed by cervical dislocation. To minimize pain during routine blood collection, mice were anesthetized with isoflurane according to approved institutional protocols.

### *Plasmodium* infections and T cell depletion

*Plasmodium chabaudi chabaudi* AS (MRA-429; MR4 Stock Center) was maintained in B6 mice. 8–12 week old female mice were used for all experiments. Infections were initiated by intraperitoneal injection of 10^6^ infected erythrocytes, and parasitemia was monitored by thin film blood smear as described [67]. To establish persistent infection in the *Ifngr1*^*-/-*^ background, mice were injected with a single 300 μg dose of α-CD4 antibody (GK1.5; generated in-house) or isotype control (LTF-2; BioXCell) 4 days post-infection (d.p.i.) with *P*. *chabaudi*. To establish persistent infection in wild-type mice, 300 μg α-CD4 was injected every 10 d from d 4 until d 64 post-infection for a total of 7 doses. All experiments in persistently infected mice were performed at least 100 d.p.i. except where noted. Simultaneously infected, isotype-treated mice were used for cleared controls. For blockade experiments, mice were treated every three days with 300 μg each α-PD-1 (RMP1-14; generated in-house) and α-LAG-3 (C9B7W; BioXCell) or with the appropriate isotype controls (HRPN and 2A3; BioXCell).

### Pharmacological treatment of infection

For subcurative drug treatment, mice were injected with a single dose of chloroquine (30 mg/kg). For rechallenge experiments, infected mice were given pyrimethamine (70 μg/mL) in drinking water for 7 d to clear infection, and clearance was confirmed by thin blood smear. Mice were re-infected with 10^6^ infected erythrocytes 60 d after cessation of pyrimethamine treatment.

### Flow cytometry

Whole blood was obtained from the submandibular vein. Red blood cells were lysed with ACK, and Fc receptors were blocked with anti-CD16/32 (2.4G2; UCSF Monoclonal Antibody Core), labeled with antibodies, and analyzed on an LSRII (BD Biosciences). Results were analyzed using FACSDiva (BD) and FlowJo (TreeStar) software. Intracellular cytokine staining was performed using the Intracellular Fixation and Permeabilization Buffer Set (eBioscience) after 6 h *in vitro* stimulation with PMA (10 ng/mL) and ionomycin (1 μg/mL) (both Fisher) in the presence of GolgiPlug (BD) and monensin (eBioscience). Antibodies to the following were used: Ly6C (clone HK1.4), CD11c (N418), CD4 (RM4.5), CD8 (53–6.7), CD11a (M17/4), CD49d (R1-2), IFNG (XMG1.2), IL-2 (JES6-5H4), PD-1 (RMP1-30), NK1.1 (PK136), Thy1.2 (53–2.1), LAG-3 (C9B7W), TCRγδ (GL-3), isotype control (eBRG1), all eBioscience; Ly6G (1A8), PD-L2 (TY25), Siglec-H (551), all Biolegend; CD11b (M1/70), F4/80 (BM8), isotype control (2A3), all UCSF Monoclonal Antibody Core; CD19 (1D3), TCRβ (H57-597), PD-L1 (10F.9G2), all Tonbo; mFCRL5 (sheep polyclonal) and CX3CR1 (goat polyclonal), R&D. Apoptotic cells were stained with propidium iodide and Annexin V (eBioscience). Leukocyte populations were delineated as follows: B cells (CD19^+^), CD8^+^ T cells (Thy1.2^+^ CD8^+^), CD4^+^ T cells (Thy1.2^+^ CD4^+^), natural killer cells (TCRβ^-^ NK1.1^+^), NKT cells (NK1.1^+^ TCRβ^+^), γδ T cells (TCRγδ^+^), granulocytes (CD11b^+^ Ly6G^+^), eosinophils (CD11b^+^ Ly6C^int^ SSC^hi^), monocytes (CD11b^+^ F4/80^int^ SSC^lo^ Ly6C as indicated), conventional DC (CD11c^hi^), and plasmacytoid DC (CD11c^int^ Siglec-H^+^). Total leukocytes were quantified on a Guava PCA System (EMD Millipore) using Viacount Reagent (EMD Millipore).

### Measurement of physiological parameters

Hematocrit was measured on 5 μL tail blood using heparinized capillary tubes. Blood glucose was monitored with a TrueResult glucometer and TrueTest strips (Nipro Diagnostics). Body temperature was measured using a Ret-3 rectal thermometer (Braintree Scientific). Mouse activity levels were quantified in an open field test [68]: briefly, mice were placed in a white 18" x 18" box and their motions were video recorded for 5 min. Data analysis was performed in the Nikon Imaging Center at UCSF/QB3 using the Tracking tool in NIS-Elements 4.20 to automatically track recorded mice. Tracking options were adjusted to ensure that the tracks accurately followed the mice, were not confused by reflections from the wall of the box, and did not stop prematurely. ALT was measured in plasma using the Alanine Transaminase Colorimetric Activity Assay Kit (Cayman Chemicals). Parasitemia and reticulocyte frequencies were enumerated from thin blood smears stained with Giemsa.

### Cytokines

Plasma IFN-γ and IL-10 were measured with the Magpix Milliplex MAP Mouse Cytokine/Chemokine Magnetic Bead Panel kit (EMD Millipore) and read on a MAGPIX instrument (Luminex).

### Tissue histology

Tissues were fixed in formalin, processed for routine sectioning, stained with hemotoxylin and eosin, and examined by an experienced anatomic pathology resident physician.

### Human monocyte analysis

Samples were obtained from established longitudinal cohorts of children in eastern Uganda, which have been described previously [21]. The mean ages of the children from low- and high-intensity areas were 5.17 years (range: 1.55–10.15) and 5.43 years (range: 0.65–10.58), respectively. Peripheral blood mononuclear cells were purified on Ficoll gradients, counted, and immediately cryopreserved in liquid nitrogen. Samples were shipped in liquid nitrogen to San Francisco, thawed in the presence of DNase, and labeled immediately with antibodies to the following: CD7 (4H9), HLA-DR (L243), CD16 (CB16), CD14 (61D3), CD19 (HIB19), all eBioscience; and CD177 (MEM-166) from Biolegend. Labeled samples were analyzed on an LSRII (BD). Classical monocytes were defined as CD177^-^CD7^-^ CD19^-^HLA^-^DR^+^CD14^hi^CD16^-^; nonclassical monocytes were defined as CD177^-^CD7^-^ CD19^-^HLA^-^DR^+^CD14^lo^CD16^+^.
